# A *NOTCH1*/*LSD1*/*BMP2* co-regulatory network mediated by miR-137 negatively regulates osteogenesis of human adipose-derived stem cells

**DOI:** 10.1186/s13287-021-02495-3

**Published:** 2021-07-22

**Authors:** Cong Fan, Xiaohan Ma, Yuejun Wang, Longwei Lv, Yuan Zhu, Hao Liu, Yunsong Liu

**Affiliations:** 1 Department of General Dentistry II, Peking University School and Hospital of Stomatology, Beijing, China; 2 National Center of Stomatology, Beijing, China; 3 National Clinical Research Center for Oral Diseases, Beijing, China; 4 National Engineering Laboratory for Digital and Material Technology of Stomatology, Beijing, China; 5 Research Center of Engineering and Technology for Computerized Dentistry Ministry of Health, Beijing, China; 6 NMPA Key Laboratory for Dental Materials, Beijing, China; 7 Department of Prosthodontics, Peking University School and Hospital of Stomatology, Beijing, China; 8 Department of Prosthodontics, Beijing Stomatological Hospital Capital Medical University, Beijing, China; 9 Central Laboratory, Peking University School and Hospital of Stomatology, Beijing, China

**Keywords:** MicroRNA, Human adipose-derived stem cells, Osteogenesis, *NOTCH1*, Signaling

## Abstract

**Background:**

MicroRNAs have been recognized as critical regulators for the osteoblastic lineage differentiation of human adipose-derived stem cells (hASCs). Previously, we have displayed that silencing of miR-137 enhances the osteoblastic differentiation potential of hASCs partly through the coordination of *lysine-specific histone demethylase 1* (*LSD1*), *bone morphogenetic protein 2* (*BMP2*), and *mothers against decapentaplegic homolog 4* (*SMAD4*). However, still numerous molecules involved in the osteogenic regulation of miR-137 remain unknown. This study aimed to further elucidate the epigenetic mechanisms of miR-137 on the osteogenic differentiation of hASCs.

**Methods:**

Dual-luciferase reporter assay was performed to validate the binding to the 3′ untranslated region (3′ UTR) of *NOTCH1* by miR-137. To further identify the role of *NOTCH1* in miR-137-modulated osteogenesis, tangeretin (an inhibitor of *NOTCH1*) was applied to treat hASCs which were transfected with miR-137 knockdown lentiviruses, then together with negative control (NC), miR-137 overexpression and miR-137 knockdown groups, the osteogenic capacity and possible downstream signals were examined. Interrelationships between signaling pathways of *NOTCH1*-*hairy and enhancer of split 1* (*HES1*), *LSD1* and *BMP2*-*SMADs* were thoroughly investigated with separate knockdown of *NOTCH1*, *LSD1*, *BMP2*, and *HES1*.

**Results:**

We confirmed that miR-137 directly targeted the 3′ UTR of *NOTCH1* while positively regulated *HES1*. Tangeretin reversed the effects of miR-137 knockdown on osteogenic promotion and downstream genes expression. After knocking down *NOTCH1* or *BMP2* individually, we found that these two signals formed a positive feedback loop as well as activated *LSD1* and *HES1*. In addition, *LSD1* knockdown induced *NOTCH1* expression while suppressed *HES1*.

**Conclusions:**

Collectively, we proposed a *NOTCH1*/*LSD1*/*BMP2* co-regulatory signaling network to elucidate the modulation of miR-137 on the osteoblastic differentiation of hASCs, thus providing mechanism-based rationale for miRNA-targeted therapy of bone defect.

**Supplementary Information:**

The online version contains supplementary material available at 10.1186/s13287-021-02495-3.

## Background

As we know, human adipose-derived stem cells (hASCs) have multilineage differentiation potentials and good application prospects for bone regeneration [1–3] because of their abundant sources and easy access for clinical uses [4]. Though plenty of signaling pathways, such as *Wnt*/*β-catenin* signal [5], *glycogen synthase kinase 3* signal [6], *bone morphogenetic protein* (*BMP*) signal [7–9], *NOTCH* signal [10, 11], and *extracellular regulated protein kinases* signal [7, 12, 13] have been demonstrated to dominate osteoblastic differentiation of hASCs, considerable research is still necessary to reveal intricate interactions between various signals and make further advances in hASC-based cell therapy.

MicroRNAs (MiRNAs) are a class of endogenous and highly conserved small non-coding single-stranded RNAs, which mediate post-transcriptional gene regulation [14] and play crucial roles in the proliferation and osteogenic differentiation of hASCs [5, 10, 15–18]. MiR-137 has manifested its anti-tumor activity in multiple malignancies, such as glioblastoma [19], melanoma [20], multiple myeloma [21], non-small cell lung cancer [22], and endometrial cancer [23]. In neural development, several studies confirm that miR-137 regulates cell proliferation, differentiation [24–28], and neuronal maturation [29–31] in adult or mouse stem cells. Nevertheless, during the process of hASCs differentiating into osteoblastic lineage, the function and epigenetic mechanisms of miR-137 have not been investigated except for our previous study [32], in which we disclose part of the mechanisms as the coordination between *lysine-specific histone demethylase 1* (*LSD1*) and *BMP2*-*mothers against decapentaplegic homolog 4* (*SMAD4*) pathway. Considering that the relationships of osteogenesis-associated signals are complex and diverse, and massive molecules participating in the *LSD1*/*BMP2*/*SMAD4* network remain unascertained, we need to further clarify the regulatory mechanisms of miR-137 on the osteogenesis.

*NOTCH* signal is a fundamental pathway in bone remodeling and skeletal homeostasis [33–35]. *Hairy and enhancer of split 1* (*HES1*), a downstream gene of *NOTCH* [36], is responsible for the actions of *NOTCH* in the skeleton, even though its osteogenic effects are cell type-specific and context-dependent. By interacting with *runt-related transcription factor 2* (*RUNX2*), *HES1* induces *osteopontin* promoter [37]. But *HES1* also binds to the *osteocalcin* (*OCN*) promoter and suppresses its transcription in osteoblastic cells [38]. *HES1* inactivation not only increases the femoral length and trabecular number in the limb bud of transgenic mice, but also enhances mineral apposition rate and suppresses bone resorption [39]. *NOTCH1* has emerged as a target of miR-137 in human renal mesangial cells [40], retinal ganglion cells [41], neurons [42], non-small cell lung cancer cells [43], and breast cancer cells [44], but whether it is directly inhibited by miR-137 has not yet been identified in hASCs. In small cell lung cancer cells, *NOTCH1* pathway is activated by *LSD1* inhibitor and suppressed due to the binding of *LSD1* [45]. Additionally, the induction of *NOTCH* signal impairs the activation of *BMP* pathway and the osteoblastic differentiation of dental follicle cells [46]. In contrast, *NOTCH1* upregulates *BMP2* expression in human aortic valve interstitial cells through the stimulation of *NF-κB* [47]. Our previous study confirmed that miR-137 knockdown induced *BMP2*-*SMAD4* pathway through the downregulation of *LSD1* dependently or independently [32], which coincides with the studies stating that *LSD1* inhibition leads to increased *BMP2* expression [48, 49]. Accordingly, we postulate a signaling network entailing *NOTCH1*-*HES1*, *LSD1*, and *BMP2*-*SMADs* pathways to unveil the miR-137 modulation on the osteogenesis of hASCs.

This study identified the interactions of miR-137 and its downstream genes and revealed that the co-regulatory signaling network of *NOTCH1*/*LSD1*/*BMP2* mediated by miR-137 negatively modulated the osteogenesis of hASCs, suggesting that miR-137 might be applied as a promising therapeutic target for bone regeneration.

## Methods

### Mice

The animal experiments were conducted in strict conformity with the guidelines of Animal Welfare Committee of Health Science Center in Peking University (LA2019019). Male, 5-week-old BALB/c-nu/nu nude mice (Charles River, Wilmington, MA, USA) were randomly assigned to 3 groups (*n* = 6 per group) and maintained with specific pathogen-free conditions.

### Cell lines

The hASCs isolated from three separate donors were purchased in ScienCell Research Laboratories (Carlsbad, CA, USA). For each donor, the in vitro cell experiments were performed at least three times individually. For proliferation culture, cells were maintained in proliferation medium (PM), containing Dulbecco’s modified Eagle’s medium (Thermo Fisher Scientific, Rockford, IL, USA), 1% (v/v) penicillin/streptomycin (Thermo Fisher Scientific) and 10% (v/v) fetal bovine serum (ExCell Bio, Shanghai, China). When the cells reached 70–80% confluence, osteoinduction was performed by adding osteogenic medium (OM), which contained the above culture medium for promoting proliferation, 100 nM dexamethasone (Sigma-Aldrich), 0.2 mM L-ascorbic acid (Sigma-Aldrich), and 10 mM β-glycerophosphate (Sigma-Aldrich). The cell culture conditions were 37 °C with 5% CO_2_ and 100% relative humidity.

### Lentivirus transfection

Recombinant lentiviruses carrying green fluorescent protein (GFP)-tagged plasmid vectors of negative control (NC), miR-137 overexpression (miR-137), miR-137 knockdown (anti-miR-137), *NOTCH1* shRNA (anti-*NOTCH1*), *LSD1* shRNA (anti-*LSD1*), *BMP2* shRNA (anti-*BMP2*), and *HES1* shRNA (anti-*HES1*) (Additional file 5: Table S1) were produced and packaged by GenePharma (Suzhou, Jiangsu, China). Lentiviral transfection of hASCs were conducted at a multiplicity of infection of 100 for 24 h with the presence of 5 mg/mL polybrene, and then selected by 1 μg/ml puromycin (Sigma-Aldrich). The transfection rates of lentiviruses were estimated by counting the number of GFP-tagged cells and total cells with an inverted fluorescence microscope (TE2000-U, Nikon, Tokyo, Japan). The hASCs transfected with miR-137 knockdown lentiviruses were seeded in 96-well plates (1 × 10^4^ cells/well) and treated with tangeretin (an inhibitor of *NOTCH1*; APExBIO, Houston, TX, USA) at a concentration of 5 μM before the examination of osteogenic differentiation ability and downstream gene expression.

### Alkaline phosphatase (ALP) staining and quantification

After 7 days of culture in PM or OM, the hASCs were used for ALP staining and activity test according to the published protocol [10]. ALP staining was operated following the BCIP/NBT staining kit (Beyotime, Shanghai, China) instructions. For the quantitative tests of ALP activities, cells were washed with phosphate buffer saline (PBS) and 1% Triton X-100 (Solarbio, Beijing, China), then scraped in milli-Q water and subjected to three cycles of freezing and thawing. By employing the BCA method and the pierce BCA protein assay kit (Thermo Fisher Scientific), total protein was read at 562 nm and computed with a bovine serum albumin standard curve according to the manufacturer’s protocol. Afterwards, ALP activity was detected at 520 nm applying an alkaline phosphatase assay kit (Jiancheng, Nanjing, Jiangsu, China) and finally normalized to the total protein concentrations of cells.

### Alizarin red S (ARS) staining and quantification

After 14 days of culture in PM or OM, the hASCs were applied to detect the matrix mineralization. Following being fixed with 95% ethanol for 30 min, cells were soaked in 1% ARS staining solution (pH 4.2; Sigma-Aldrich, St. Louis, MO, USA) for 20 min at room temperature. To assess the degree of mineralization, stained areas of each well were separately dissolved in 100 mM cetylpyridinium chloride (Sigma-Aldrich) for 1 h and the absorbances were detected at 562 nm. Finally, the relative ARS intensity was normalized to the total protein concentrations of cells.

### RNA extraction, reverse transcription, and quantitative real-time polymerase chain reaction (qRT-PCR)

After 3, 7, and 14 days of culture in PM or OM respectively, total RNA of cells was isolated with TRIzol (Invitrogen, Carlsbad, CA, USA) and synthesized into the first-strand cDNA using a reverse transcription system (Takara, Tokyo, Japan). All the transcripts were quantified using the FastStart universal SYBR green master (ROX) (Roche, Indianapolis, IN, USA) and a 7500 real-time PCR detection system (Applied Biosystems, Foster City, CA, USA). Relative expression levels of mRNA and miRNA were normalized to *GAPDH* mRNA and *U6* snRNA, respectively. The sequences of the primers employed are listed in Additional file 6: Table S2.

### Western blotting

The hASCs were rinsed with ice PBS three times and immersed in RIPA buffer (HuaxingBio, Beijing, China) mixed with protease inhibitor cocktail (HuaxingBio). The pierce BCA protein assay kit (Thermo Fisher Scientific) was used to determine the protein concentration. A 25-μg sample of protein was added and separated by 10% sodium dodecyl sulfate-polyacrylamide gel electrophoresis, then followed by transfer to the polyvinylidene difluoride membranes (Millipore, Bedford, MA, USA). Strips on the membranes were blocked with 5% nonfat dry milk (BioRuler, Danbury, CT, USA) for 1 h at room temperature, incubated overnight at 4 °C with primary antibodies at a dilution of 1:1000, and then for 1 h at room temperature with goat anti-rabbit secondary antibodies labeled with horseradish peroxidase (ZSGB-BIO, Beijing, China; ZB-2301) at a dilution of 1:10,000. The primary antibodies used were as follows: anti-GAPDH (ZSGB-BIO; TA-08), anti-NOTCH1 (Cell Signaling Technology, Beverly, MA, USA; 3608S), anti-HES1(Abcam, Cambridge, UK; ab108937), anti-LSD1 (Cell Signaling Technology; 2139S), anti-BMP2 (Abcam; ab14933), anti-SMAD4 (Abcam; ab40759), anti-phosphorylated SMAD1/5 (anti-p-SMAD1/5; Cell Signaling Technology; 9516S), and anti-RUNX2 (Cell Signaling Technology; 12556). Relative band intensities were measured with the ImageJ software.

### Dual-luciferase reporter assay

The 3′ untranslated region (3′ UTR) alignments of the target regions in *NOTCH1* were predicted by TargetScan and RNA22. Reporter vectors were constructed based on the previous method [32]. The 3′ UTR sequences of *NOTCH1*, which contained the possible binding sites of miR-137, were PCR amplified and then inserted into pEZX-MT06 vectors (GeneCopoeia, Rockville, MD, USA) to create *NOTCH1*-WT (wild-type *NOTCH1*) luciferase reporter plasmids. Mutated forms were generated by site-directed mutagenesis (GeneCopoeia) and named *NOTCH1*-MT (mutant-type *NOTCH1*) luciferase reporter plasmids. For luciferase assay, the hASCs were planted on 24-well culture plates with a density of 5 × 10^4^/well and co-transfected with 1 μg *NOTCH1*-WT or *NOTCH1*-MT plasmids, 100 nM NC or miR-137 mimics, and lipofectamine 3000 (Invitrogen). The luciferase activities were examined by a dual-luciferase reporter assay system (Promega, Madison, WI, USA) 48 h later, and standardized to renilla luciferase activity for each transfected well.

### Heterotopic osteogenesis examinations *in vivo*

After the transfection with NC, miR-137 and anti-miR-137 lentiviruses, hASCs of the third passage were maintained in PM for 1 week, collected, and incubated with auto-setting calcium phosphate cement (ACPC; Rebone, Shanghai, China) for 1 h at 37 °C. Then the hASC-ACPC mixtures were transplanted subcutaneously to the dorsal regions of nude mice (*n* = 6 per group) for the analyses of heterotopic bone formation in vivo 8 weeks later. After being collected and fixed in 4% paraformaldehyde, the samples were photographed with soft X-ray. The radiograph was obtained by applying a Senograph 2000D molybdenum-rhodium twin target X-ray apparatus (GE, Fairfield, CT, USA). The radiation distance is 200 mm and the radiographing conditions were 22.0 kV, 35.0 mA. For histological evaluation, the specimens were decalcified in 10% ethylene diamine tetraacetic acid solution (pH 7.4) for 14 days, embedded into paraffin, then sliced into 5 μm-thick sections before the subsequent hematoxylin and eosin (HE) staining and Masson trichrome staining. The rabbit anti-OCN primary antibodies diluted to 1:100 (Servicebio, Wuhan, Hubei, China; GB11233) were used for immunohistochemical (IHC) staining.

### Statistical analysis

Data and statistical analyses were conducted with SPSS Statistics 20.0 software (IBM, Armonk, NY, USA). All data were shown as mean ± standard deviation (SD) of three individual experiments. For the comparison of two independent or multiple groups, Mann-Whitney *U* test or Kruskal-Wallis test were applied, respectively. A two-tailed test with *p* value < 0.05 was indicated as statistically significant.

## Results

### MiR-137 reversely regulates hASC differentiation along osteoblastic lineage in vitro

Our previous study displayed an overall downward expression trend of miR-137 in hASCs during the osteoblastic induction and identified its negative role in this biological process [32]. Since lacking other studies on the osteogenic function of miR-137, we re-verified the reliability of our previous results. After transfecting hASCs with lentiviruses of NC, miR-137 overexpression, and miR-137 knockdown (Additional file 1: Fig. S1a), we evaluated the transfection rate was over 90% by computing the percentage of GFP-tagged cells (Additional file 1: Fig. S1b). Meanwhile, the transfection effects were quantitatively determined on 3 days, 7 days, and 14 days by qRT-PCR analysis (Additional file 1: Fig. S1c).

ALP staining and activity assays displayed that miR-137 overexpression reduced ALP activity of hASCs under proliferation condition or osteogenic induction, but miR-137 knockdown reversed the effects observed with miR-137 overexpression (Additional file 2: Fig. S2a, b). ARS staining and quantification were applied to test the calcium deposits of extracellular matrix. More mineralized nodules were presented in miR-137 knockdown group while less in miR-137 overexpression group when both were compared with NC group (Additional file 2: Fig. S2c, d). Besides, the characteristic genes expressed in different stages of osteogenesis, including *RUNX2*, *ALP*, and *OCN*, were examined by qRT-PCR and presented significant decreases in miR-137 overexpression group but dramatically increased in miR-137 knockdown group (Additional file 2: Fig. S2e). According to these data, we substantiated that miR-137 inhibits in vitro osteoblastic activity of hASCs.

### MiR-137 reversely regulates hASC differentiation along osteoblastic lineage *in vivo*

In order to validate in vivo osteogenic effects of miR-137, hASCs were transfected with NC, miR-137, and anti-miR-137 lentiviruses and separately mixed with ACPC, and then the compounds were subcutaneously implanted into the dorsum of nude mice (Additional file 3: Fig. S3a). After 8 weeks, the total volume and mean density of the harvested samples were assessed and manifested an apparent enhancement in miR-137 knockdown group but remarkable reduction in miR-137 overexpression group (Additional file 3: Fig. S3b, c).

Histological analyses of bone formation were performed by staining of HE, Masson trichrome, and IHC staining for OCN. HE staining showed more new bone formation in miR-137 knockdown group when comparing with NC group, which displayed only a very small amount of osteoid, but we could hardly observe any new bone or osteoid in miR-137 overexpression group. Similarly, thicker and more compact blue-green-stained collagen fiber bundles were detected in miR-137 knockdown group than in another two groups, but overexpression of miR-137 led to the thinnest collagen deposition. Moreover, we found that dark-brown stained OCN granules were the most widespread in the cells of miR-137 knockdown group, fewer in NC group, and none could be discerned in miR-137 overexpression group (Additional file 3: Fig. S3d). Consequently, in accordance with the results of in vitro experiments, miR-137 subdues the osteoblastic activity of hASCs in vivo.

### MiR-137 regulates *NOTCH1*-*HES1* pathway by directly targeting *NOTCH1*

**Fig. 1:**
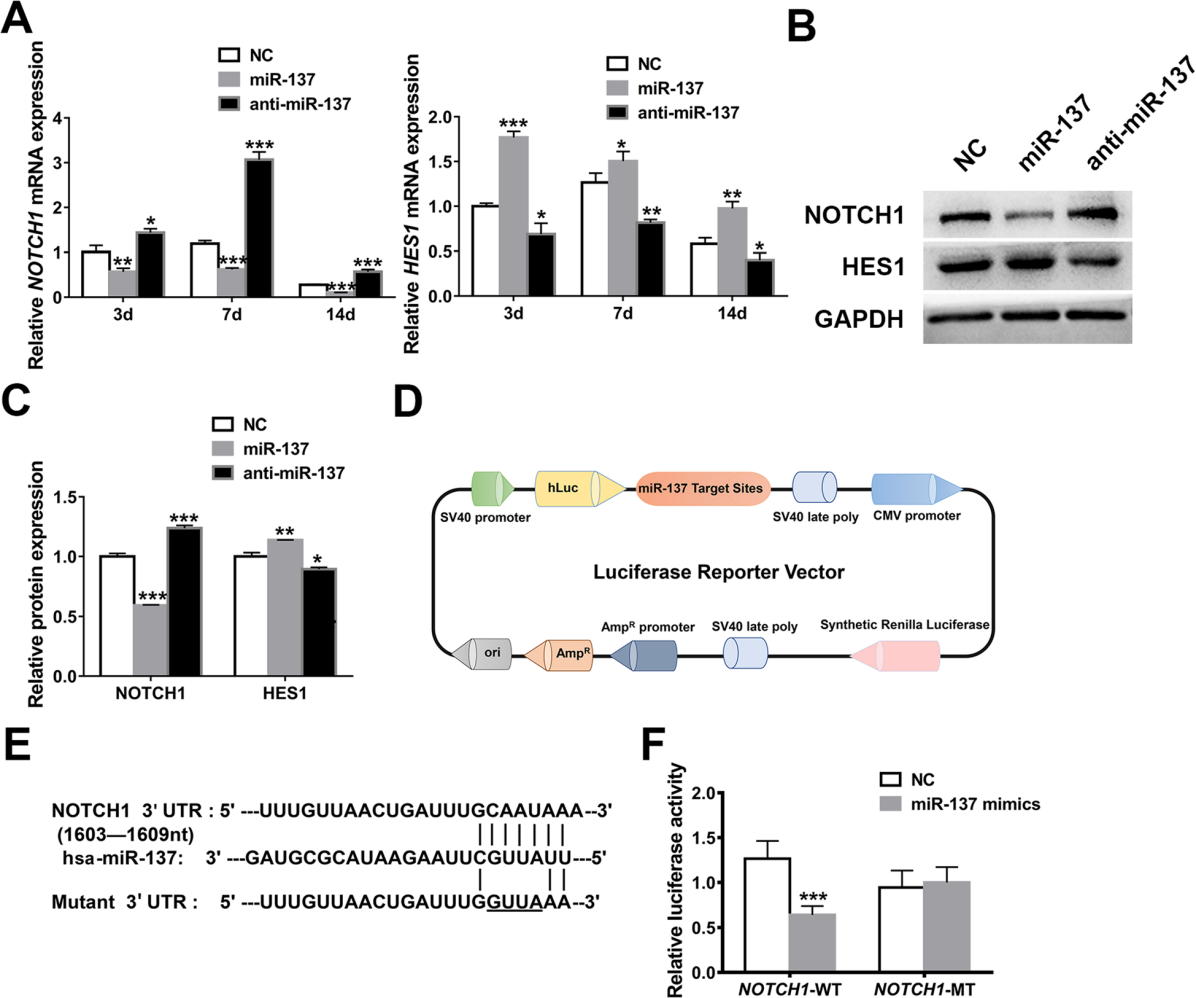
MiR-137 regulates *NOTCH1*-*HES1* pathway by directly targeting *NOTCH1*. **a** Relative expression analyses of *NOTCH1* and *HES1* by qRT-PCR in transfected hASCs on 3 days, 7 days, and 14 days. **b**, **c** Western blotting (**b**) and band intensity analyses (**c**) of NOTCH1 and HES1 in transfected hASCs on 7 days. **d** The structure diagram of a luciferase reporter vector containing the 3′ UTR of *NOTCH1*-WT or *NOTCH1*-MT. **e** Softwares (TargetScan and RNA22) predicted the binding sites of miR-137 located in the 3′ UTR of *NOTCH1*-WT (underlined parts indicated the mutated bases in *NOTCH1*-MT). **f** Luciferase activity analyses of the *NOTCH1*-WT and *NOTCH1*-MT. Data are shown as mean ± SD of three independent experiments performed in triplicate. *p < 0.05, **p < 0.01, ***p < 0.001 versus respective NC group

To ascertain the influences of miR-137 on *NOTCH1* pathway, we first examined the expression of *NOTCH1* and its downstream signal *HES1* with miR-137 overexpression or knockdown. When compared with NC group, the mRNA and protein levels of *NOTCH1* showed obvious increase in miR-137 knockdown group while marked reduction in miR-137 overexpression group. Contrary to *NOTCH1*, the expression tendency of *HES1* accorded with the changes of miR-137 (Fig. 1a–c). These findings suggested that *NOTCH1* is negatively regulated while *HES1* is positively regulated by miR-137.

To further identify whether miR-137 could directly bind to *NOTCH1* in hASCs as it does in other cell lines [40–44], dual-luciferase reporter assays were carried out. The presumed targeting sites of miR-137 in the 3′ UTR of *NOTCH1* were forecasted by two prediction softwares (TargetScan and RNA22). Then the luciferase reporter vectors carrying the 3′ UTR of *NOTCH1*-WT or *NOTCH1*-MT were constructed (Fig. 1d, e) and the relative luciferase activities were detected. MiR-137 mimics significantly repressed the luciferase activity in *NOTCH1*-WT group while they had no significant influences in *NOTCH1*-MT group when both groups were compared with their respective NC group (Fig. 1f). These results validated that miR-137 directly binds to the 3′ UTR of *NOTCH1* and induces the expression of *HES1* in hASCs.

### *NOTCH1* inhibitor reverses the effects of miR-137 knockdown on osteogenesis and downstream genes expression

**Fig. 2:**
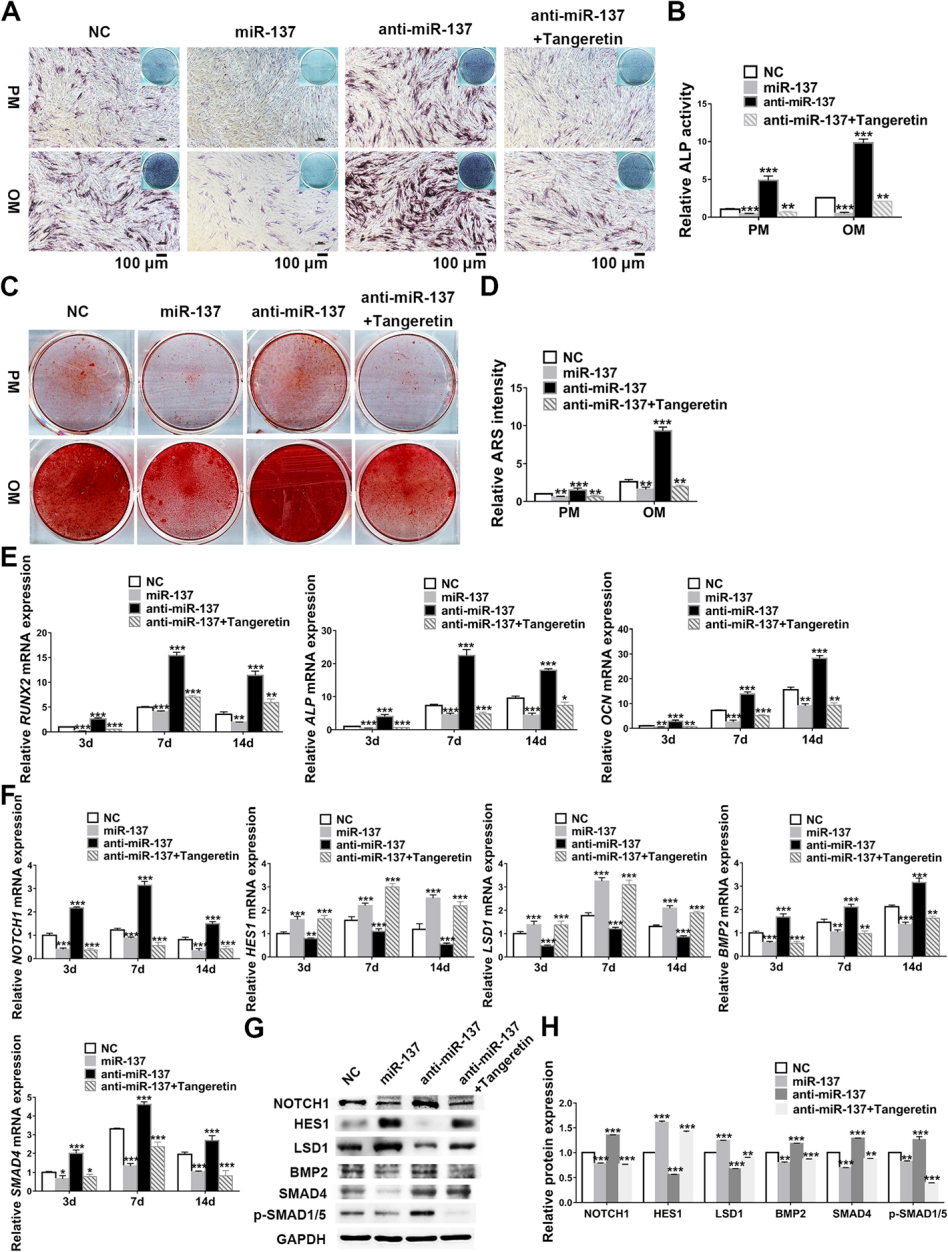
*NOTCH1* inhibitor (tangeretin) reverses the effects of miR-137 knockdown on osteogenesis and downstream genes expression. **a**, **b** ALP staining (**a**) and quantification (**b**) of transfected hASCs after a 7-day culture in PM or OM (scale bar = 100 μm). **c**, **d** ARS staining (**c**) and quantification (**d**) of transfected hASCs after a 14-day culture in PM or OM. **e** Relative expression analyses of *RUNX2*, *ALP*, and *OCN* by qRT-PCR in transfected hASCs on 3 days, 7 days, and 14 days. **f** Relative expression analyses of *NOTCH1*, *HES1*, *LSD1*, *BMP2*, and *SMAD4* by qRT-PCR in transfected hASCs on 3 days, 7 days, and 14 days. **g**, **h** Western blotting (**g**) and band intensity analyses (**h**) of NOTCH1, HES1, LSD1, BMP2, SMAD4, and p-SMAD1/5 in transfected hASCs on 7 days. Data are shown as mean ± SD of three independent experiments performed in triplicate. *p < 0.05, **p < 0.01, ***p < 0.001 versus respective NC group

To confirm whether miR-137 regulated osteogenesis through *NOTCH1*, we employed *NOTCH1* inhibitor (tangeretin) in hASCs transfected with miR-137 knockdown lentiviruses. In vitro osteogenic stainings and quantification manifested that when compared with NC group, miR-137 knockdown evidently strengthened ALP activity and extracellular matrix calcification, whereas the promoted osteogenic ability caused by miR-137 knockdown were completely reversed by tangeretin treatment, and miR-137 overexpression group displayed impaired osteogenic differentiation potential as previously mentioned (Fig. 2a–d). In accord with the results of osteogenic stainings, qRT-PCR detection showed that the expression of *RUNX2*, *ALP*, and *OCN* were enhanced in miR-137 knockdown group while reduced in miR-137 overexpression group, but the addition of tangeretin abrogated the induction of these genes by miR-137 knockdown and even suppressed their expression (Fig. 2e).

To further authenticate whether *NOTCH1* mediated the mechanisms of the osteogenic regulation by miR-137, we examined the expression of miR-137 downstream signals in the above four groups. Both in miR-137 overexpression and tangeretin-treated groups, *NOTCH1* was significantly repressed, though miR-137 knockdown induced its expression. The mRNA and protein expression showed that *HES1* and *LSD1* changed synchronously with the alterations of miR-137, but tangeretin-treated group inverted the downregulation of these two genes by miR-137 knockdown when compared with NC group. In addition, the mRNA or protein expression levels of *BMP2*, *SMAD4*, and p-SMAD1/5 showed that these genes were induced by miR-137 knockdown while inhibited by miR-137 overexpression. Similarly, tangeretin inhibited the activation of *BMP2*-*SMADs* pathway initiated by miR-137 knockdown (Fig. 2f–h). Combined with the above results, we affirmed that *NOTCH1* inhibitor reverses the osteogenic modulation effects of miR-137 knockdown.

### *NOTCH1* knockdown impairs osteogenesis by inducing *HES1*

**Fig. 3:**
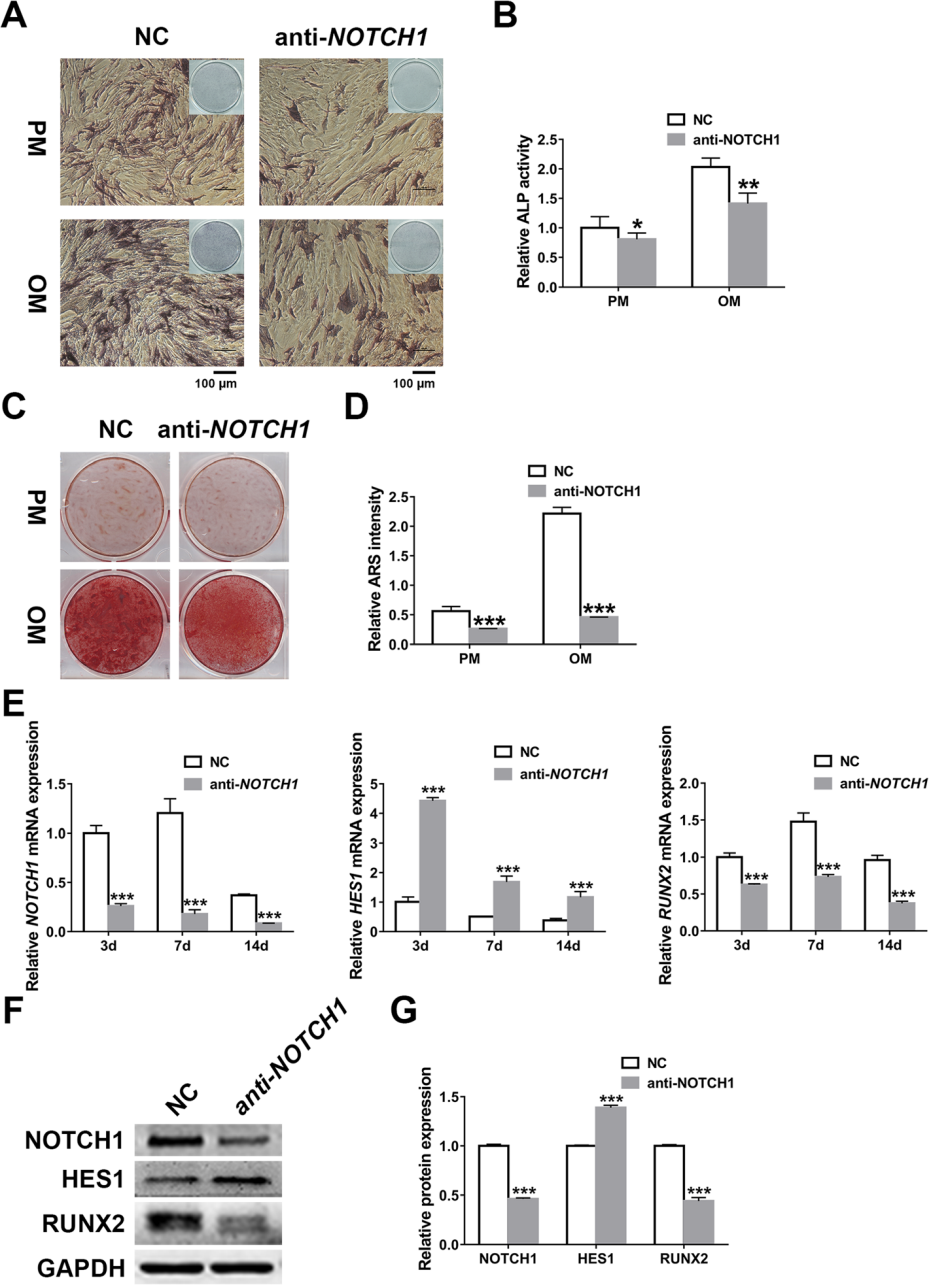
*NOTCH1* knockdown impairs osteogenesis by inducing *HES1*. **a**, **b** ALP staining (**a**) and quantification (**b**) of transfected hASCs after a 7-day culture in PM or OM (scale bar = 100 μm). **c**, **d** ARS staining (**c**) and quantification (**d**) of transfected hASCs after a 14-day culture in PM or OM. **e** Relative expression analyses of *NOTCH1*, *HES1*, and *RUNX2* by qRT-PCR in transfected hASCs on 3 days, 7 days, and 14 days. **f**, **g** Western blotting (**f**) and band intensity analyses (**g**) of NOTCH1, HES1, and RUNX2 in transfected hASCs on 7 days. Data are shown as mean ± SD of three independent experiments performed in triplicate. *p < 0.05, **p < 0.01, ***p < 0.001 versus respective NC group

To determine the influences of *NOTCH1* knockdown on the osteoblastic potential of hASCs, we applied ALP and ARS stainings combined with quantitative analysis and found that *NOTCH1* knockdown attenuated ALP activity and extracellular mineralization (Fig. 3a–d). As the downstream molecules of *NOTCH1*, *HES1*, and *RUNX2* were further detected at mRNA and protein levels in hASCs transfected with *NOTCH1* knockdown lentiviruses. Coincident with the impacts of miR-137 on *NOTCH1*-*HES1* pathway, *NOTCH1* knockdown induced the expression of *HES1* while repressed *RUNX2* (Fig. 3e–g). Our results indicated that *NOTCH1* knockdown impedes the osteogenic potential of hASCs by the stimulation of *HES1*, corroborating the former conclusions that miR-137 inhibits osteogenesis by the downregulation of *NOTCH1* and upregulation of *HES1*.

**Fig. 4:**
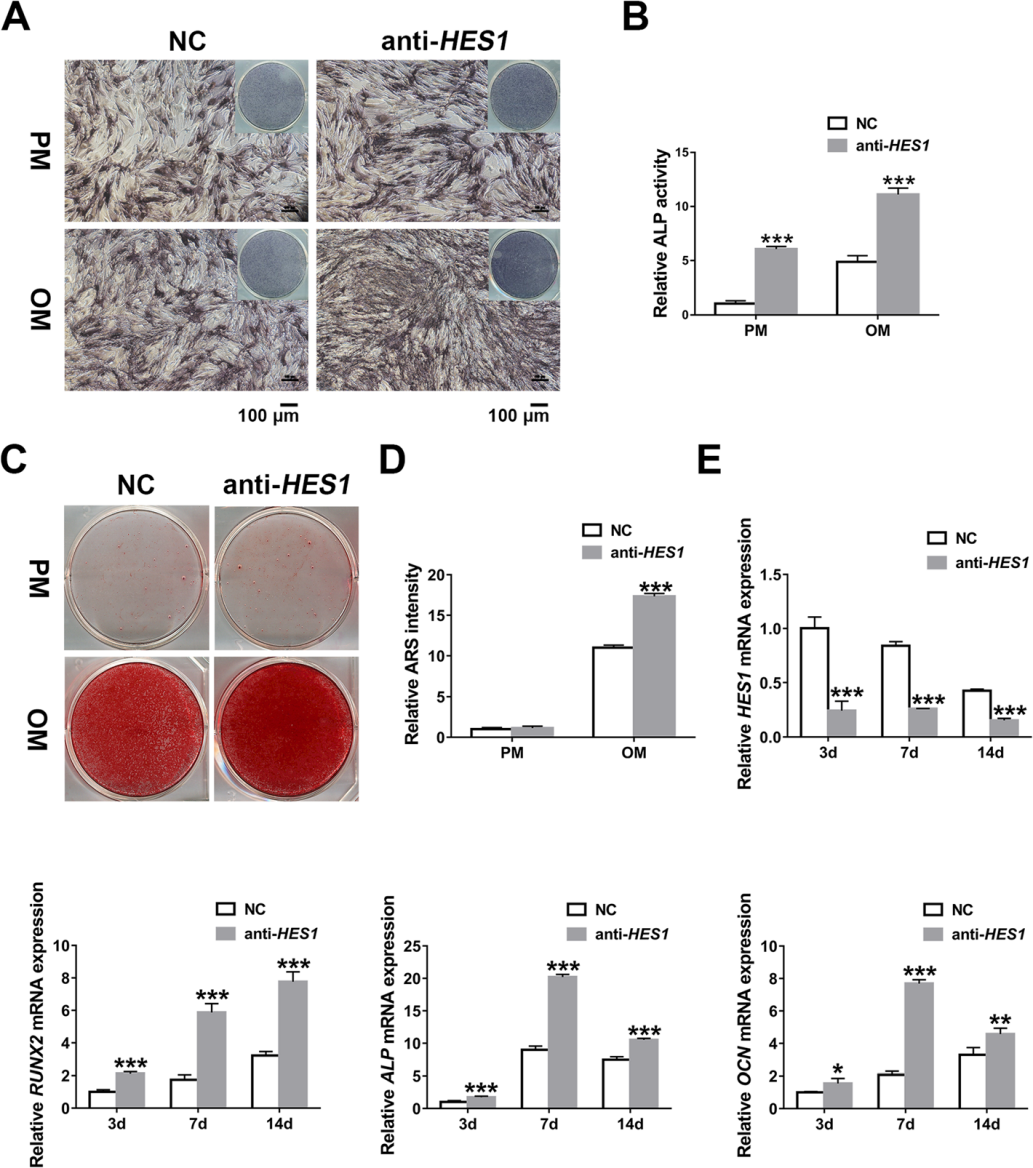
*HES1* knockdown promotes osteogenesis of hASCs in vitro. **a**, **b** ALP staining (**a**) and quantification (**b**) of transfected hASCs after a 7-day culture in PM or OM (scale bar = 100 μm). **c**, **d** ARS staining (**c**) and quantification (**d**) of transfected hASCs after a 14-day culture in PM or OM. **e** Relative expression analyses of *HES1*, *RUNX2*, *ALP*, and *OCN* by qRT-PCR in transfected hASCs on 3 days, 7 days, and 14 days. Data are shown as mean ± SD of three independent experiments performed in triplicate. *p < 0.05, **p < 0.01, ***p < 0.001 versus respective NC group

Then we checked the impacts of *HES1* on the osteogenic differentiation of hASCs. After knocking down *HES1*, we examined the osteogenic ability by the application of osteogenic stainings and quantification as well as qRT-PCR detection of *RUNX2*, *ALP*, and *OCN*. All these in vitro osteogenic tests showed promoted osteogenic potential of hASCs with *HES1* knockdown (Fig. 4). Therefore, we demonstrated that *HES1* plays a negative role in the osteoblastic differentiation of hASCs and intervenes in *NOTCH1*-induced osteogenesis.

### *NOTCH1* knockdown induces *LSD1* while inhibits *BMP2*-*SMADs* pathway

**Fig. 5:**
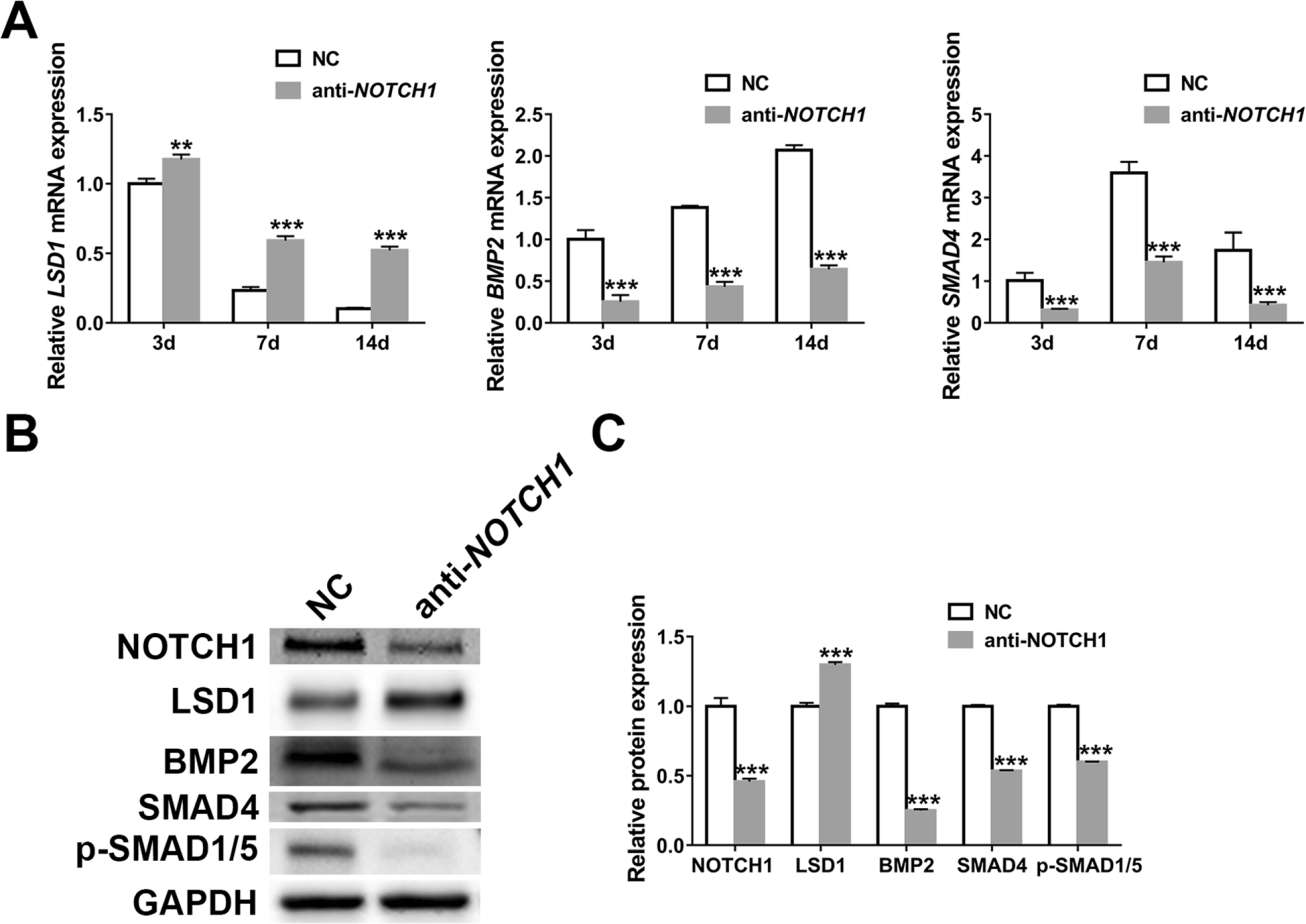
*NOTCH1* knockdown induces *LSD1* while inhibits *BMP2*-*SMADs* pathway. **a** Relative expression analyses of *LSD1*, *BMP2*, and *SMAD4* by qRT-PCR in transfected hASCs on 3 days, 7 days, and 14 days. **b**, **c** Western blotting (**b**) and band intensity analyses (**c**) of NOTCH1, LSD1, BMP2, SMAD4, and p-SMAD1/5 in transfected hASCs on 7 days. Data are shown as mean ± SD of three independent experiments performed in triplicate. *p < 0.05, **p < 0.01, ***p < 0.001 versus respective NC group

Since our previous study has demonstrated that miR-137 upregulated *LSD1* while downregulated *BMP2* and *SMAD4* in hASCs [32], we further investigated the influences of *NOTCH1* knockdown on *LSD1* and *BMP2*-*SMADs* pathway. As predicted, the expression of *LSD1* significantly increased after knocking down *NOTCH1* while *BMP2*, *SMAD4*, and p-SMAD1/5 decreased apparently (Fig. 5). Therefore, we deduced that the osteogenic inhibition of *NOTCH1* knockdown is also dependent on the activation of *LSD1* and suppression of *BMP2*-*SMADs* pathway.

### *LSD1* knockdown regulates *NOTCH1*-*HES1* pathway

**Fig. 6:**
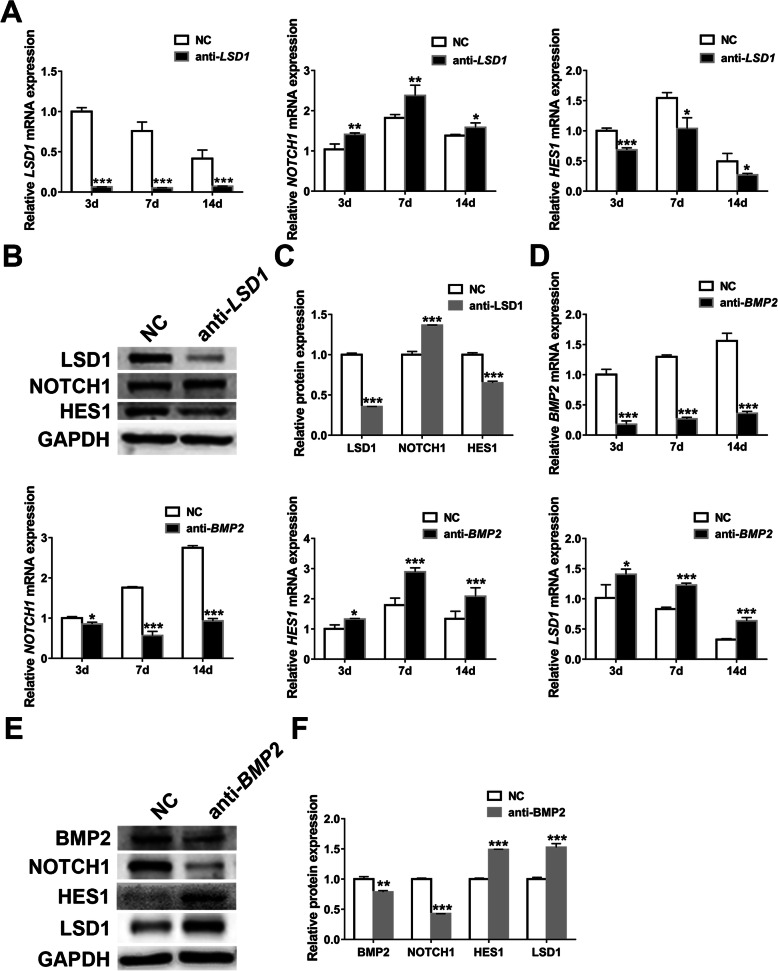
*LSD1* or *BMP2* knockdown influences *NOTCH1*/*LSD1*/*BMP2* signaling network. **a** Relative expression analyses of *LSD1*, *NOTCH1*, and *HES1* by qRT-PCR in transfected hASCs on 3 days, 7 days, and 14 days. **b**, **c** Western blotting (**b**) and band intensity analyses (**c**) of LSD1, NOTCH1, and HES1 in transfected hASCs on 7 days. **d** Relative expression analyses of *BMP2*, *NOTCH1*, *HES1*, and *LSD1* by qRT-PCR in transfected hASCs on 3 days, 7 days, and 14 days. **e**, **f** Western blotting (**e**) and band intensity analyses (**f**) of BMP2, NOTCH1, HES1, and LSD1 in transfected hASCs on 7 days. Data are shown as mean ± SD of three independent experiments performed in triplicate. *p < 0.05, **p < 0.01, ***p < 0.001 versus respective NC group

As above, we have affirmed that *NOTCH1* acted as a negative regulator in *LSD1* expression. But considering the complex interplay of signaling molecules, we tried to clarify whether *LSD1* had feedback effects on *NOTCH1*-*HES1* pathway. Notably, we found that *LSD1* knockdown led to a higher level of *NOTCH1* while lowered the expression of *HES1* when compared with NC group (Fig. 6a–c), thus prompting a reciprocal negative relationship between *NOTCH1* and *LSD1*.

### *BMP2* knockdown inhibits *HOTCH1* while induces *HES1* and *LSD1*

To gain further insights into the relationships between *NOTCH1*, *LSD1*, and *BMP2* signals, we then examined the expression of *NOTCH1*, *HES1*, and *LSD1* at mRNA and protein levels after knocking down *BMP2*. Importantly, the expression of *NOTCH1* dramatically decreased while *HES1* and *LSD1* increased with *BMP2* knockdown (Fig. 6d–f). Combining with the above results that *NOTCH1* knockdown inhibited *BMP2*-*SMADs* pathway, we verified a positive feedback loop between *NOTCH1* and *BMP2*. Furthermore, our previous study revealed that silencing of *LSD1* promoted the osteoblastic potential of hASCs by stimulating *BMP2*-*SMAD4* signaling pathway [32]. Here again, we observed upward tendencies in the expression of *BMP2*, *SMAD4*, *RUNX2*, and *ALP* with *LSD1* knockdown (Additional file 4: Fig. S4). Therefore, a negative interplay between *LSD1* and *BMP2* was also confirmed by us.

## Discussion

Dysfunction of miR-137 has been implicated in several cancer types such as glioblastoma [19], melanoma [20], multiple myeloma [21], non-small cell lung cancer [22], and endometrial cancer [23]. In addition, miR-137 has been found to play an essential role in neural development and maturation, with several studies displaying an association with cell proliferation and neurogenic differentiation [24–31]. However, little is known concerning its functions and regulatory mechanisms on the osteoblastic differentiation, especially in mesenchymal stem cells. Silencing of miR-137-3p is found to facilitate the osteogenesis of bone marrow-derived mesenchymal stem cells by targeting *RUNX2* [50]. Previously, we demonstrated that silencing of miR-137 promoted the osteoblastic activity in hASCs and revealed its modulation on the signaling network of *LSD1*/*BMP2*/*SMAD4* as part of the mechanisms [32]. Interestingly, our former research confirmed a positive role of miR-137 in *LSD1* expression, which was contrary to several studies reporting that miR-137 directly binds to *LSD1* [23, 26, 51, 52]. The contradicted outcomes might be associated with the various biological features of different cell types, and we deduced that there probably exist intermediary regulators working between miR-137 and *LSD1* during the osteogenesis of hASCs. Notably, this study identified that *NOTCH1* was a direct target of miR-137 in hASCs, and *NOTCH1*-*HES1* pathway was engaged in the crosstalk between *LSD1* and *BMP2*-*SMADs* pathway. In this way, *NOTCH1* signal mediated the control of miR-137 on *LSD1*/*BMP2*/*SMAD4* network. Moreover, the interrelations of the above signals were validated comprehensively and a *NOTCH1*/*LSD1*/*BMP2* co-regulatory network was established, further elucidating the epigenetic mechanisms of miR-137 during the process of hASCs differentiating into osteoblastic lineage.

After reconfirming the inhibitory impacts of miR-137 on the osteoblastic activity of hASCs both in vitro and in vivo, we demonstrated that miR-137 negatively regulated the expression of *NOTCH1* while positively regulated *HES1*. *NOTCH* signaling pathway influences tumorigenesis as well as embryonic development [53] because of its crucial role in cell fate determination, proliferation, differentiation, and apoptosis [54]. Though it is still debatable whether *NOTCH* signal serves as a positive or negative regulator in osteogenesis [55], our data displayed impaired osteogenic capacity of hASCs after knocking down *NOTCH1*. Most noteworthy, *NOTCH1* was validated as a direct target gene of miR-137 in hASCs, the same as in other cell lines [40–44]. *HES1* is known as a potential downstream gene of *NOTCH1* in many studies, but it is not affected in *NOTCH1* knockout mice while the expression of *HES5* and *HES-related repressor protein* (*HERP*)*1*, *-2*, and *-3* are greatly diminished [56–58]. As a transcriptional regulator in the *NOTCH* signaling pathway, *recombination signal binding protein* (*RBPJ*) gene disruption in homozygous mice exhibits reduced *HES5* expression, but not for *HES1* [56]. Given the various effects on *OCN*, *osteopontin*, and *RUNX2* [37, 38], *HES1* influences the osteogenesis inconsistently depending on the different cellular environments. This study exhibited enhanced osteogenic differentiation of hASCs with *HES1* knockdown. Moreover, *NOTCH1* knockdown upregulated *HES1*, indicating that *NOTCH1* acts as a negative regulator in the expression of *HES1*. To further identify whether *NOTCH1* mediated the osteogenic differentiation of hASCs modulated by miR-137, we applied *NOTCH1* inhibitor (tangeretin) in hASCs transfected with miR-137 knockdown lentiviruses and found that tangeretin reversed the impacts of miR-137 knockdown on osteogenesis and downstream genes expression, thus verifying the vital role of *NOTCH1* in the osteogenic regulation of miR-137. Collectively, we brought insight into how the *NOTCH1*-*HES1* pathway was influenced and involved in the osteogenic modulation of miR-137.

*LSD1* has been linked to the repression of *NOTCH1* pathway in various cell types [45, 59–63], though one study states that it functions as a corepressor when associated with *RBPJ*-repressor complex and as a *NOTCH1* coactivator upon *NOTCH* activation [64]. Nevertheless, few studies have reported the interplay between *NOTCH1* and *LSD1* during the osteogenesis of hASCs and whether this interaction contributes to the osteogenic control of miR-137 is still unknown. Coincident with the influences of miR-137 on *NOTCH1* and *LSD1*, we uncovered a negative interaction between *NOTCH1* and *LSD1* with separate knockdown of them. More intriguingly, despite inducing the expression of *NOTCH1*, *LSD1* knockdown repressed *HES1*. Thus, the opposite expression trends of *NOTCH1* and *HES1* caused by *LSD1* knockdown might reinforce the downregulation of *HES1* by *NOTCH1* alone. These results authenticated the crosstalk between *NOTCH1*-*HES1* pathway and *LSD1*, through which miR-137 regulates the osteogenic differentiation of hASCs.

*BMP* signal is a canonical pathway in skeleton and *BMP2*-*SMAD4* pathway has been shown to participate in the osteogenic regulation of miR-137 by us [32]. After knocking down *NOTCH1* or *BMP2* individually, we observed suppressed expression of *NOTCH1*, *BMP2*, and *SMADs* simultaneously, indicating a positive interrelationship between *NOTCH1* and *BMP2*-*SMADs* pathway. Besides, increased *HES1* expression with *BMP2* knockdown proved the negative effects of *NOTCH1* on *HES1* from another aspect. Considering our previous results that *LSD1* knockdown activated the *BMP2*-*SMAD4* pathway [32], in turn, we investigated the impacts of *BMP2* on *LSD1*. As expected, *LSD1* was significantly upregulated with *BMP2* knockdown. Thus, these findings suggested a negative feedback loop between *LSD1* and *BMP2*-*SMADs* pathway.

**Fig. 7:**
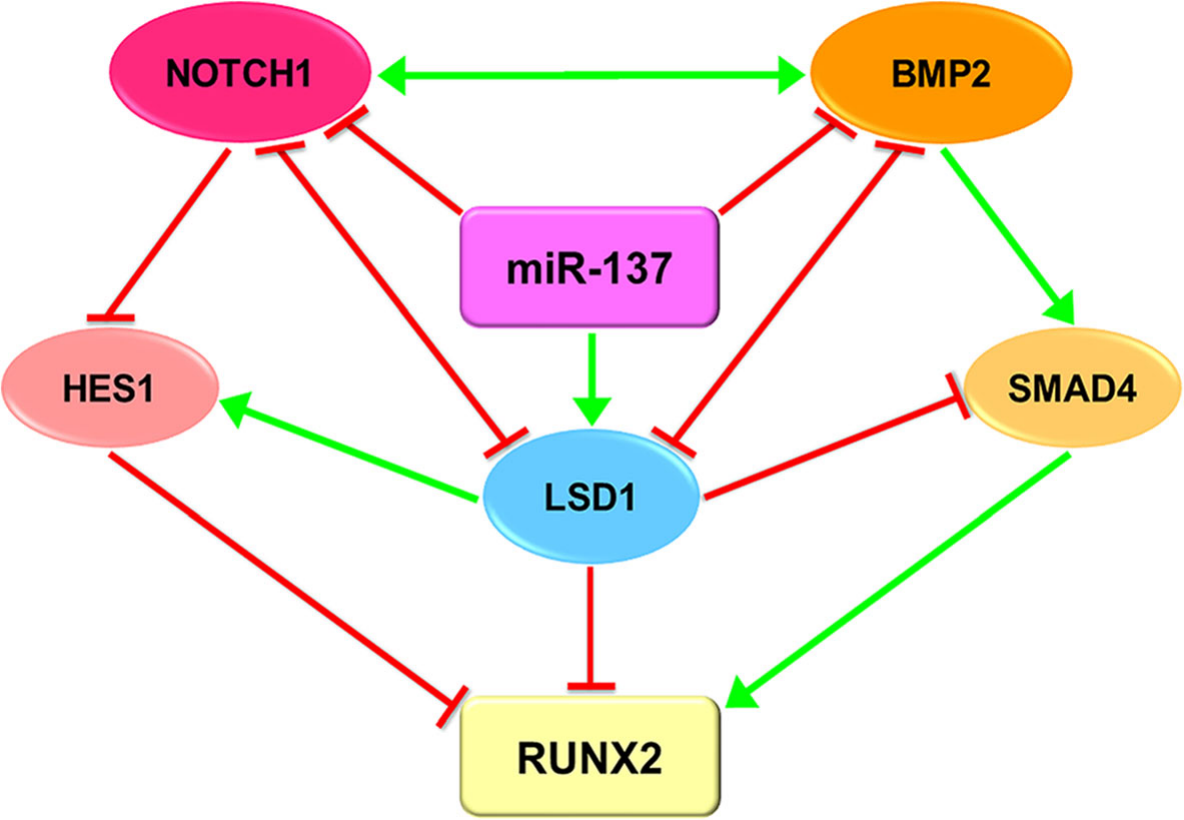
A diagrammatic view of *NOTCH1*/*LSD1*/*BMP2* signaling network in miR-137-mediated differentiation of hASCs towards osteoblastic lineage. In this complex network model, miR-137 modulates *NOTCH1*-*HES1*, *LSD1*, and *BMP2*-*SMADs* pathways simultaneously. Depending on the positive feedback loop between *NOTCH1* and *BMP2*, as well as the negative reciprocal relationship between *LSD1* and *NOTCH1* or *BMP2*, the impacts of miR-137 on the above three signaling pathways are strengthened and the *NOTCH1*/*LSD1*/*BMP2* network regulates the osteogenesis of hASCs coordinately (red lines represent downregulation and green lines represent upregulation)

In conclusion, our results revealed that depending on the reciprocal negative regulation between *NOTCH1* and *LSD1*, *LSD1* and *BMP2*, as well as the synergistic function between *NOTCH1* and *BMP2*, miR-137 negatively regulates osteogenesis of hASCs through the *NOTCH1*/*LSD1*/*BMP2* co-regulatory signaling network (Fig. 7).

## Conclusions

In summary, our study provided a relatively comprehensive rationale for the negative modulation of miR-137 during the hASC differentiation towards osteoblastic lineage and established a co-regulatory network of *NOTCH1*/*LSD1*/*BMP2* to elucidate the underlying mechanisms, which is of substantial importance for potential targeted therapy of bone-related diseases.

## Supplementary Information

**Additional file 1: Figure S1.** Efficiency determination of lentiviral transfection. a The structure diagram of packaged lentiviruses. b Microscopic images of transfected hASCs with GFP-tagged lentiviruses under the ordinary (left panel) and fluorescent light (right panel). Scale bar = 100 μm. c Relative expression analysis of miR-137 by qRT-PCR in transfected hASCs on 3 d, 7 d and 14 d. Data are shown as mean ± SD of three independent experiments performed in triplicate. *p < 0.05, **p < 0.01, ***p < 0.001 versus respective NC group.

**Additional file 2: Figure S2.** MiR-137 reversely regulates hASC differentiation along osteoblastic lineage *in vitro*. a, b ALP staining (a) and quantification (b) of transfected hASCs after a 7-day culture in PM or OM (scale bar = 100 μm). c, d ARS staining (c) and quantification (d) of transfected hASCs after a 14-day culture in PM or OM. e Relative expression analyses of *RUNX2*, *ALP* and *OCN* by qRT-PCR in transfected hASCs on 3 d, 7 d and 14 d. Data are shown as mean ± SD of three independent experiments performed in triplicate. *p < 0.05, **p < 0.01, ***p < 0.001 versus respective NC group.

**Additional file 3: Figure S3.** MiR-137 reversely regulates hASC differentiation along osteoblastic lineage *in vivo*. a Schema of the experimental design for *in vivo* study. *n* = 6 per group. b Representative soft X-ray photographs of the specimens which were subcutaneously harvested from the dorsal pockets of nude mice 8 weeks later. c Mean density analyses by the application of ImageJ software. Data are shown as mean ± SD of six independent experiments. *p < 0.05, **p < 0.01, ***p < 0.001 versus NC group. d Heterotopic bone formation was evaluated by histological stainings: HE, Masson trichrome staining (scale bar = 50 μm), and IHC staining of OCN (scale bar = 20 μm). Typical dark brown particles indicating OCN depositions in hASCs were marked with black arrows.

**Additional file 4: **Figure S4. *LSD1* knockdown activates *BMP2*-*SMAD4* pathway and osteogenesis-associated genes expression. a Relative expression analyses of *BMP2*, *SMAD4*, *RUNX2* and *ALP* by qRT-PCR in transfected hASCs on 3 d, 7 d and 14 d. b, c Western blotting (b) and band intensity analyses (c) of LSD1, BMP2, SMAD4 and RUNX2 in transfected hASCs on 7 d. Data are shown as mean ± SD of three independent experiments performed in triplicate. *p < 0.05, **p < 0.01, ***p < 0.001 versus respective NC group.

**Additional file 5: Table S1.** Sequences for lentiviral vectors.

**Additional file 6: Table S2.** Sequences of the primers for qRT-PCR.

**Additional file 7: Table S3.** Key resources table.

## Data Availability

The datasets used and analyzed during the current study are available from the corresponding author on reasonable request.

## Abbreviations

3′ UTR: 3′ untranslated region
ACPC: Auto-setting calcium phosphate cement
ALP: Alkaline phosphatase
ARS: Alizarin red S
*BMP*: Bone morphogenetic protein
GFP: Green fluorescent protein
hASCs: Human adipose-derived stem cells
HE: Hematoxylin and eosin
*HES1*: Hairy and enhancer of split 1
IHC: Immunohistochemical
LSD1: Lysine-specific histone demethylase 1
miRNA: MicroRNA
OCN: Osteocalcin
OM: Osteogenic medium
PM: Proliferation medium
qRT-PCR: Quantitative real-time polymerase chain reaction
RUNX2: Runt-related transcription factor 2
SMAD4: Mothers against decapentaplegic homolog 4
p-SMAD1/5: Phosphorylated SMAD1/5
