# Supplementary material for: A NOTCH1/LSD1/BMP2 co-regulatory network mediated by miR-137 negatively regulates osteogenesis of human adipose-derived stem cells
Source: Stem Cell Res Ther. 2021 Jul 22;12:417. doi: 10.1186/s13287-021-02495-3 (PMC8296522; doi:10.1186/s13287-021-02495-3)
Supplement: Supplementary file 1 — Additional file 1: Figure S1. Efficiency determination of lentiviral transfection. a The structure diagram of packaged lentiviruses. b Microscopic images of transfected hASCs with GFP-tagged lentiviruses under the ordinary (left panel) and fluorescent light (right panel). Scale bar = 100 μm. c Relative expression analysis of miR-137 by qRT-PCR in transfected hASCs on 3 d, 7 d and 14 d. Data are shown as mean ± SD of three independent experiments performed in triplicate. *p < 0.05, **p < 0.01, ***p < 0.001 versus respective NC group. [file 13287_2021_2495_MOESM1_ESM.pdf]

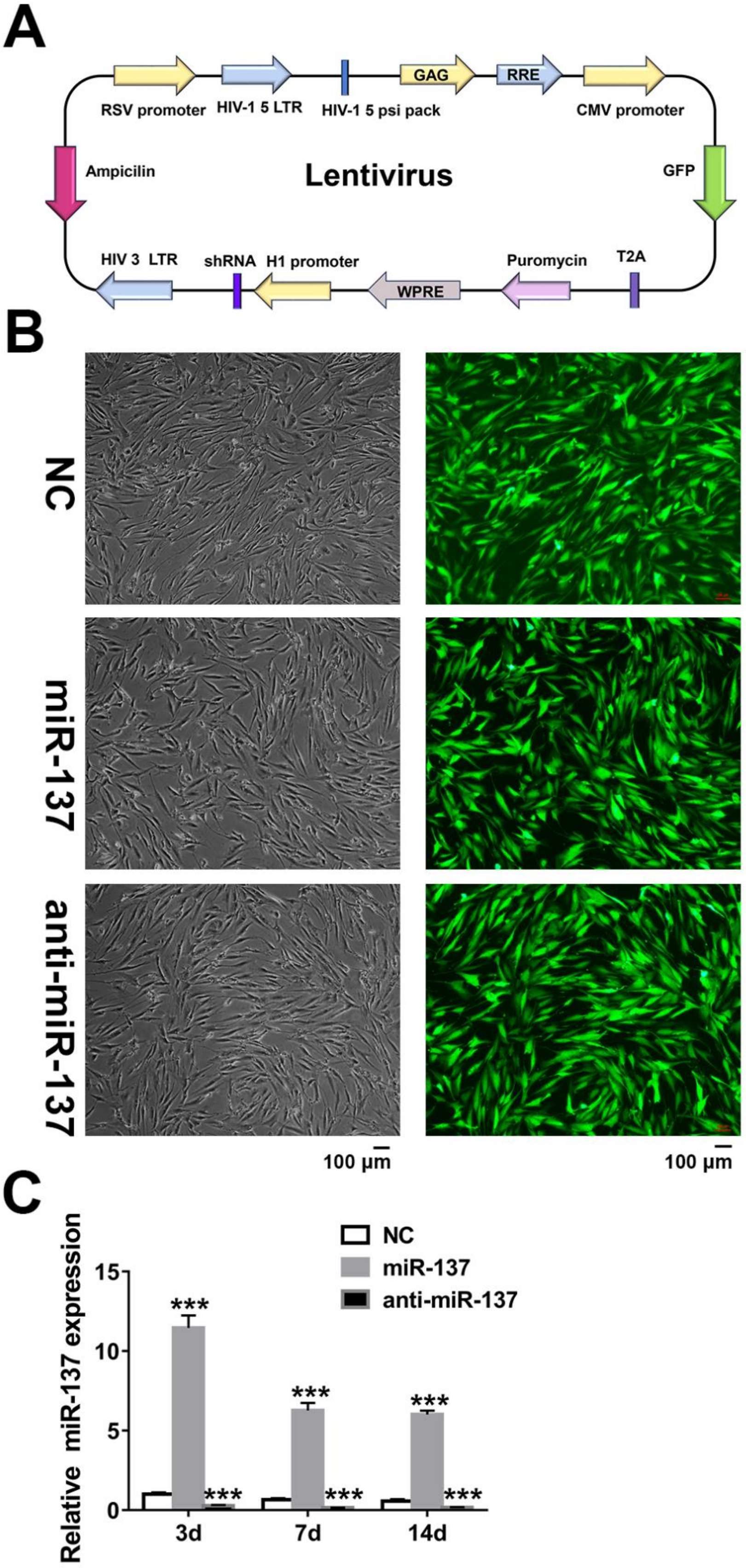
A
RSV promoter
HIV-1 5 LTR
HIV-1 5 psi pack
GAG
RRE
CMV promoter
Ampicilin
Lentivirus
GFP
HIV 3 LTR
shRNA
H1 promoter
WPRE
Puromycin
T2A
B
NC
miR-137
anti-miR-137
100 μm
100 μm
C
NC
miR-137
anti-miR-137
Relative miR-137 expression
15
10
5
0
***
***
***
***
***
***
3d
7d
14d

Additional file 1: Figure S1. Efficiency determination of lentiviral transfection. **a** The structure diagram of packaged lentiviruses. **b** Microscopic images of transfected hASCs with GFP-tagged lentiviruses under the ordinary (left panel) and fluorescent light (right panel). Scale bar = 100 μm. **c** Relative expression analysis of miR-137 by qRT-PCR in transfected hASCs on 3 d, 7 d and 14 d. Data are shown as mean ± SD of three independent experiments performed in triplicate. *$p < 0.05$, **$p < 0.01$, ***$p < 0.001$ versus respective NC group.
