# Supplementary material for: A NOTCH1/LSD1/BMP2 co-regulatory network mediated by miR-137 negatively regulates osteogenesis of human adipose-derived stem cells
Source: Stem Cell Res Ther. 2021 Jul 22;12:417. doi: 10.1186/s13287-021-02495-3 (PMC8296522; doi:10.1186/s13287-021-02495-3)
Supplement: Supplementary file 2 — Additional file 2: Figure S2. MiR-137 reversely regulates hASC differentiation along osteoblastic lineage in vitro. a, b ALP staining (a) and quantification (b) of transfected hASCs after a 7-day culture in PM or OM (scale bar = 100 μm). c, d ARS staining (c) and quantification (d) of transfected hASCs after a 14-day culture in PM or OM. e Relative expression analyses of RUNX2, ALP and OCN by qRT-PCR in transfected hASCs on 3 d, 7 d and 14 d. Data are shown as mean ± SD of three independent experiments performed in triplicate. *p < 0.05, **p < 0.01, ***p < 0.001 versus respective NC group. [file 13287_2021_2495_MOESM2_ESM.pdf]

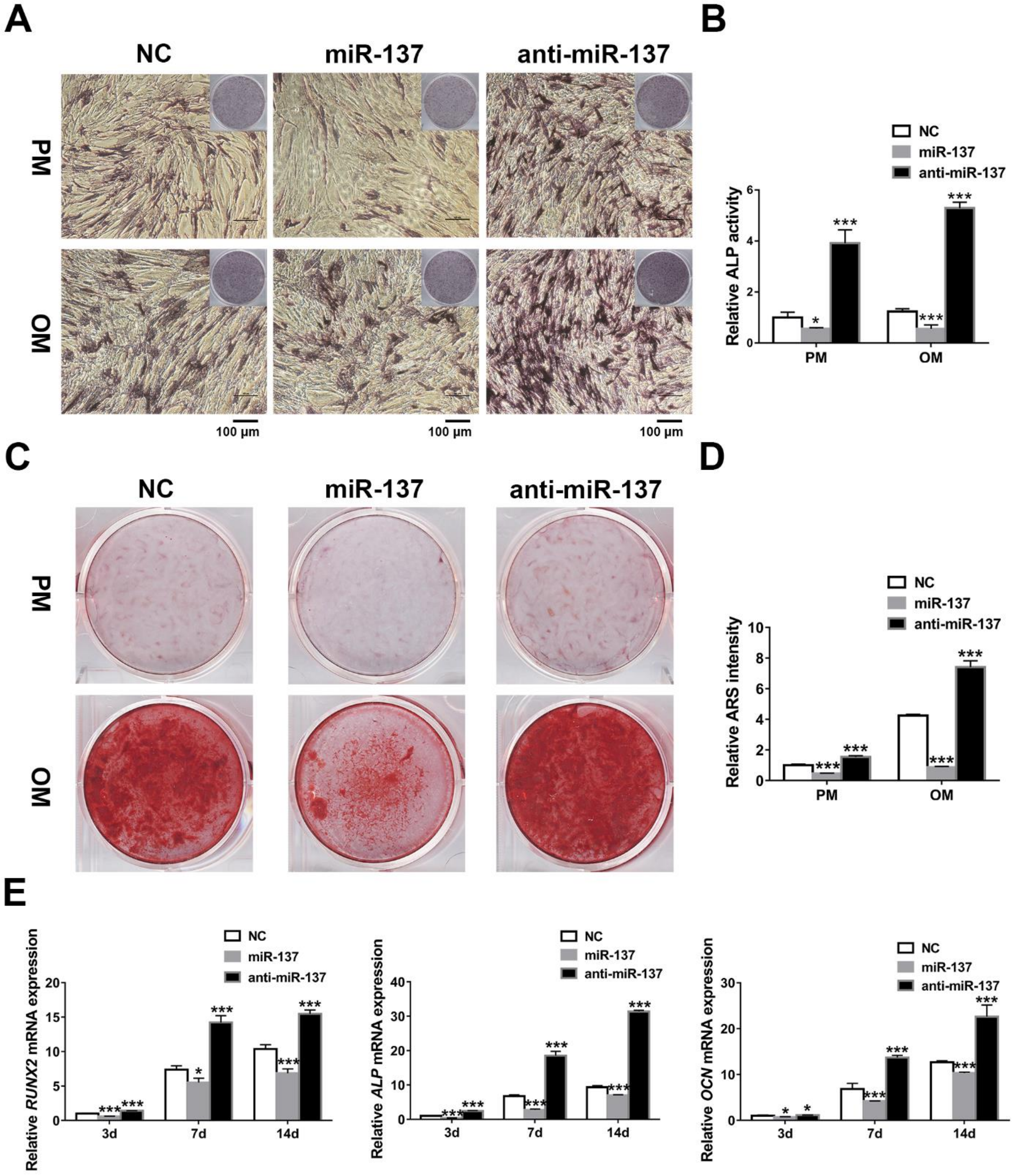

Additional file 2: Figure S2. MiR-137 reversely regulates hASC differentiation along osteoblastic lineage *in vitro*. **a**, **b** ALP staining (**a**) and quantification (**b**) of transfected hASCs after a 7-day culture in PM or OM (scale bar = 100 μm). **c**, **d** ARS staining (**c**) and quantification (**d**) of transfected hASCs after a 14-day culture in PM or OM. **e** Relative expression analyses of *RUNX2*, *ALP* and *OCN* by qRT-PCR in transfected

hASCs on 3 d, 7 d and 14 d. Data are shown as mean ± SD of three independent experiments performed in triplicate. *$p < 0.05$, **$p < 0.01$, ***$p < 0.001$ versus respective NC group.
