# Supplementary material for: A NOTCH1/LSD1/BMP2 co-regulatory network mediated by miR-137 negatively regulates osteogenesis of human adipose-derived stem cells
Source: Stem Cell Res Ther. 2021 Jul 22;12:417. doi: 10.1186/s13287-021-02495-3 (PMC8296522; doi:10.1186/s13287-021-02495-3)
Supplement: Supplementary file 3 — Additional file 3: Figure S3. MiR-137 reversely regulates hASC differentiation along osteoblastic lineage in vivo. a Schema of the experimental design for in vivo study. n = 6 per group. b Representative soft X-ray photographs of the specimens which were subcutaneously harvested from the dorsal pockets of nude mice 8 weeks later. c Mean density analyses by the application of ImageJ software. Data are shown as mean ± SD of six independent experiments. *p < 0.05, **p < 0.01, ***p < 0.001 versus NC group. d Heterotopic bone formation was evaluated by histological stainings: HE, Masson trichrome staining (scale bar = 50 μm), and IHC staining of OCN (scale bar = 20 μm). Typical dark brown particles indicating OCN depositions in hASCs were marked with black arrows. [file 13287_2021_2495_MOESM3_ESM.pdf]

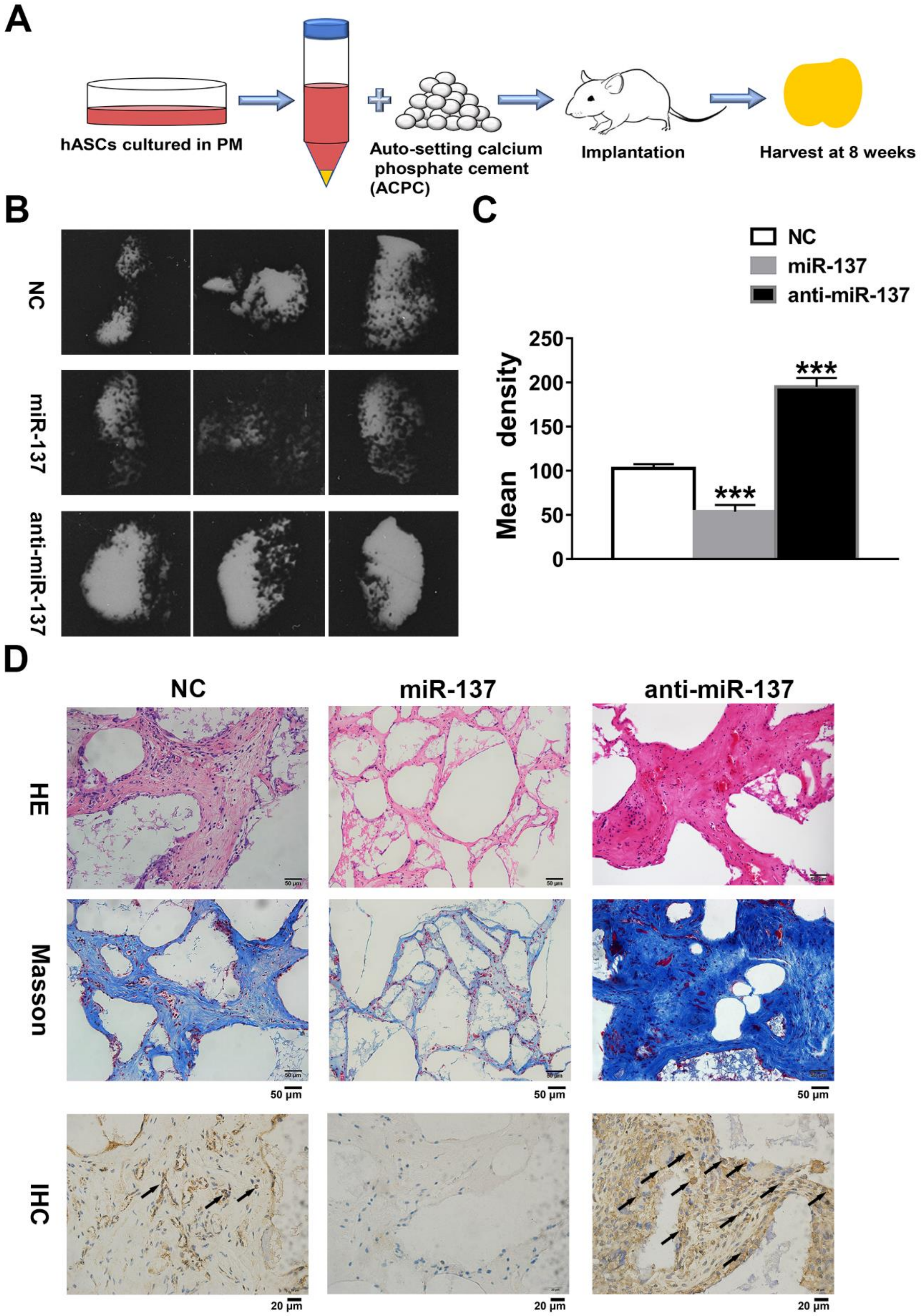

Additional file 3: Figure S3. MiR-137 reversely regulates hASC differentiation along osteoblastic lineage *in vivo*. **a** Schema of the experimental design for *in vivo* study. n = 6 per group. **b** Representative soft X-ray photographs of the specimens which were

subcutaneously harvested from the dorsal pockets of nude mice 8 weeks later. **c** Mean density analyses by the application of ImageJ software. Data are shown as mean ± SD of six independent experiments. *$p < 0.05$, **$p < 0.01$, ***$p < 0.001$ versus NC group. **d** Heterotopic bone formation was evaluated by histological stainings: HE, Masson trichrome staining (scale bar = 50 μm), and IHC staining of OCN (scale bar = 20 μm). Typical dark brown particles indicating OCN depositions in hASCs were marked with black arrows.
