# Supplementary material for: A NOTCH1/LSD1/BMP2 co-regulatory network mediated by miR-137 negatively regulates osteogenesis of human adipose-derived stem cells
Source: Stem Cell Res Ther. 2021 Jul 22;12:417. doi: 10.1186/s13287-021-02495-3 (PMC8296522; doi:10.1186/s13287-021-02495-3)
Supplement: Supplementary file 4 — Additional file 4: Figure S4. LSD1 knockdown activates BMP2-SMAD4 pathway and osteogenesis-associated genes expression. a Relative expression analyses of BMP2, SMAD4, RUNX2 and ALP by qRT-PCR in transfected hASCs on 3 d, 7 d and 14 d. b, c Western blotting (b) and band intensity analyses (c) of LSD1, BMP2, SMAD4 and RUNX2 in transfected hASCs on 7 d. Data are shown as mean ± SD of three independent experiments performed in triplicate. *p < 0.05, **p < 0.01, ***p < 0.001 versus respective NC group. [file 13287_2021_2495_MOESM4_ESM.pdf]

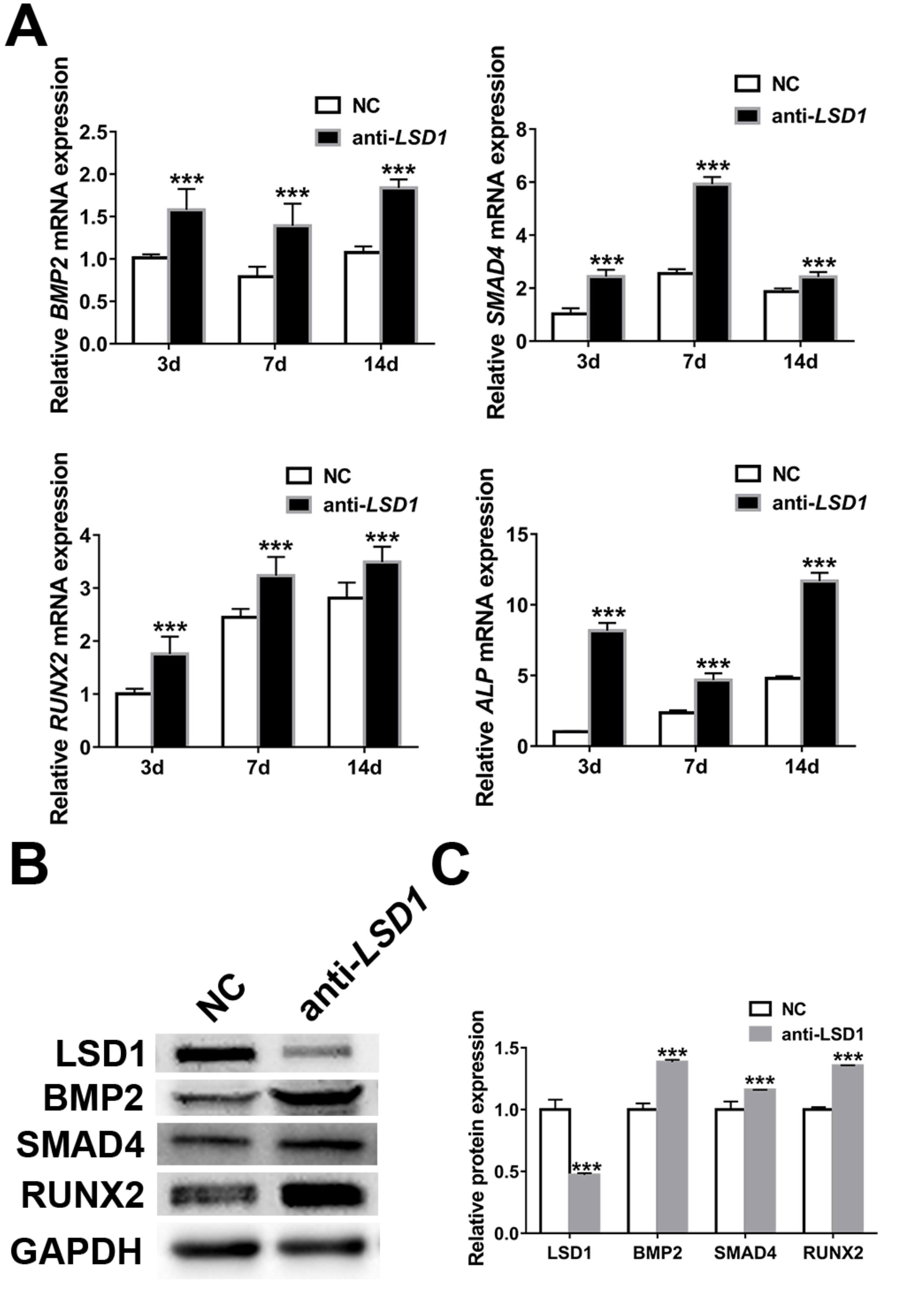

Additional file 4: Figure S4. *LSD1* knockdown activates *BMP2-SMAD4* pathway and

osteogenesis-associated genes expression. **a** Relative expression analyses of *BMP2*, *SMAD4*, *RUNX2* and *ALP* by qRT-PCR in transfected hASCs on 3 d, 7 d and 14 d. **b**, **c** Western blotting (**b**) and band intensity analyses (**c**) of LSD1, BMP2, SMAD4 and RUNX2 in transfected hASCs on 7 d. Data are shown as mean ± SD of three independent experiments performed in triplicate. *$p < 0.05$, **$p < 0.01$, ***$p < 0.001$ versus respective NC group.
