# Supplementary material for: A NOTCH1/LSD1/BMP2 co-regulatory network mediated by miR-137 negatively regulates osteogenesis of human adipose-derived stem cells
Source: Stem Cell Res Ther. 2021 Jul 22;12:417. doi: 10.1186/s13287-021-02495-3 (PMC8296522; doi:10.1186/s13287-021-02495-3)
Supplement: Supplementary file 5 — Additional file 5: Table S1. Sequences for lentiviral vectors. [file 13287_2021_2495_MOESM5_ESM.docx]

**Additional file 5: Table S1.** Sequences for lentiviral vectors.

| **Name** | **Sequence (5′ to 3′)** |
| --- | --- |
| NC | TTCTCCGAACGTGTCACGT |
| miR-137 | TTATTGCTTAAGAATACGCGTAG |
| anti-miR-137 | CTACGCGTATTCTTAAGCAATAA |
| anti-*NOTCH1* | CCATGGTACCAATCATGAA |
| anti-*LSD1* | GCACCTTATAACAGTGATACT |
| anti-*BMP2* | GCAGTTTCCATCACCGAATTA |
| anti-*HES1* | GCCAGTTTGCTTTCCTCATTC |
