# Supplementary material for: A NOTCH1/LSD1/BMP2 co-regulatory network mediated by miR-137 negatively regulates osteogenesis of human adipose-derived stem cells
Source: Stem Cell Res Ther. 2021 Jul 22;12:417. doi: 10.1186/s13287-021-02495-3 (PMC8296522; doi:10.1186/s13287-021-02495-3)
Supplement: Supplementary file 6 — Additional file 6: Table S2. Sequences of the primers for qRT-PCR. [file 13287_2021_2495_MOESM6_ESM.docx]

**Additional file 6: Table S2.** Sequences of the primers for qRT-PCR.

| **Gene** | **Sequence (5'-3')** | | **Length (bp)** | **GenBank Accession No.** |
| --- | --- | --- | --- | --- |
|  | **Forward primer** | **Reverse primer** |  |  |
| *NOTCH1* | CGCTGACGGAGTACAAGTG | GTAGGAGCCGACCTCGTTG | 245 | NM_017617 |
| *HES1* | TCAACACGACACCGGATAAAC | GCCGCGAGCTATCTTTCTTCA | 153 | NM_005524 |
| *LSD1* | TGACCGGATGACTTCTCAAGA | GTTGGAGAGTAGCCTCAAATGTC | 155 | NM_001009999 |
| *BMP2* | ACCCGCTGTCTTCTAGCGT | TTTCAGGCCGAACATGCTGAG | 180 | NM_001200 |
| *SMAD4* | ACGAACGAGTTGTATCACCTGG | TGCACGATTACTTGGTGGATG | 173 | NM_005359 |
| *RUNX2* | TGGTTACTGTCATGGCGGGTA | TCTCAGATCGTTGAACCTTGCTA | 101 | NM_001015051 |
| *ALP* | AACATCAGGGACATTGACGTG | GTATCTCGGTTTGAAGCTCTTCC | 159 | NM_001127501 |
| *OCN* | GGCGCTACCTGTATCAATGG | GTGGTCAGCCAACTCGTCA | 110 | NM_199173 |
| *GAPDH* | ACAACTTTGGTATCGTGGAAGG | GCCATCACGCCACAGTTTC | 101 | NM_001256799 |
