# Supplementary material for: A NOTCH1/LSD1/BMP2 co-regulatory network mediated by miR-137 negatively regulates osteogenesis of human adipose-derived stem cells
Source: Stem Cell Res Ther. 2021 Jul 22;12:417. doi: 10.1186/s13287-021-02495-3 (PMC8296522; doi:10.1186/s13287-021-02495-3)
Supplement: Supplementary file 7 — Additional file 7: Table S3. Key resources table. [file 13287_2021_2495_MOESM7_ESM.docx]

**Additional file 7: Table S3.** Key resources table.

| **Reagent or resource** | **Source** | **Identifier** |
| --- | --- | --- |
| **Antibodies** | | |
| Anti-RUNX2 | Cell Signaling Technology | Cat#12556; RRID: AB_2732805 |
| Anti-NOTCH1 | Cell Signaling Technology | Cat#3608S; RRID: AB_2153354 |
| Anti-HES1 | Abcam | Cat#ab108937; RRID: AB_10862625 |
| Anti-LSD1 | Cell Signaling Technology | Cat#2139S; RRID: AB_2070135 |
| Anti-BMP2 | Abcam | Cat#ab14933; RRID: AB_2243574 |
| Anti-SMAD4 | Abcam | Cat#ab40759; RRID: AB_777980 |
| Anti-(p) SMAD1/5 | Cell Signaling Technology | Cat#9516S; RRID: AB_491015 |
| Anti-GAPDH | ZSGB-BIO | Cat#TA-08 |
| Anti-rabbit IgG, HPR-linked antibody | ZSGB-BIO | Cat#ZB-2301 |
| Anti-mouse IgG, HPR-linked antibody | ZSGB-BIO | Cat#ZB-230 |
| Anti-OCN | Servicebio | Cat#GB11233 |
| **Chemicals, peptides, and recombinant proteins** | | |
| DMEM | Thermo Fisher Scientific | Cat#111995065 |
| FBS | ExCell Bio | Cat#FND500 |
| Trypsin-EDTA | Thermo Fisher Scientific | Cat#25200056 |
| Antibiotic (Penicillin/Streptomycin) | Thermo Fisher Scientific | Cat#15140122 |
| DMSO | Sigma-Aldrich | Cat#D2650 |
| Puromycin | Sigma-Aldrich | Cat#P8833 |
| Lipofectamine 3000 | Invitrogen | Cat#L3000015 |
| PBS | Solarbio | Cat#P1010 |
| L-ascobic acid | Sigma-Aldrich | Cat#A4544 |
| β-glycerophosphate disodium salt hydrate | Sigma-Aldrich | Cat#G9422 |
| Dexamethasone | Sigma-Aldrich | Cat#D1756 |
| Tangeretin | APExBIO | Cat#N2077 |
| TRIzol^TM^ | Invitrogen | Cat#15596018 |
| RIPA buffer | HuaxingBio | Cat#HX1862 |
| Protease inhibitor cocktail | HuaxingBio | Cat#HX1863 |
| Triton X-100 | Solarbio | Cat#T8200 |
| TE buffer | Solarbio | Cat#T1120 |
| DNase/RNase-free water | Solarbio | Cat#R1600 |
| LB agar | Sigma-Aldrich | Cat# L2897 |
| Tween 20 | Solarbio | Cat#T8220 |
| 10% SDS solution | Solarbio | Cat#S1010 |
| Glycine | BioRuler | Cat#RJ0507 |
| Tris | BioRuler | Cat#RJ0507 |
| Sodium chloride | Hushi | Cat#10019318 |
| Non-fat powdered milk | BioRuler | Cat#RH61873 |
| ColorMixed protein marker | Solarbio | Cat#PR1920 |
| FastStart universal SYBR green master (ROX) | Roche | Cat#04913914001 |
| Alizarin red s | Sigma-Aldrich | Cat#A5533 |
| Auto-setting calcium phosphate cement | Rebone | Cat#RB-SK-005G |
| **Critical commercial assays** | | |
| BCIP/NBT alkaline phosphatase color development kit | Beyotime | Cat#C3206 |
| Alkaline phosphatase assay kit | Jiancheng | Cat#A059-2-2 |
| Pierce™ BCA protein assay kit | Thermo Fisher Scientific | Cat#23227 |
| PrimeScript™ RT reagent kit | Takara | Cat#RR037A |
| eECL western blot kit | CWbiotech | Cat#CW0049M |
| Dual-luciferase reporter assay system | Promega | Cat#E1910 |
| **Experimental models: cell lines** | | |
| Human adipose-derived stem cells | ScienCell Research Laboratories | Cat#7510 (8278, 19382, 11537) |
| **Experimental models: organisms/strains** | | |
| 5-week BALB/c nude mice | Charles River | N/A |
| **Oligonucleotides** | | |
| Sequences for Lentiviral Vector, see Table S1 | This paper | N/A |
| Primers for qRT-PCR, see Table S2 | This paper | N/A |
| **Recombinant DNA** | | |
| pGLV3/H1/GFP + puro vector | Genepharma | N/A |
| pEZX-MT06 vector | GeneCopoeia | N/A |
| **Software and algorithms** | | |
| SPSS statistics 20.0 | IBM | https://www.ibm.com/cn-zh/analytics/spss-statistics-software |
| ImageJ | NIH | https://imagej.nih.gov/ij/index.html |
| GraphPad Prism v.6 | GraphPad Software | https://www.graphpad.com/scientific-software/prism/ |
